# Transcriptome Analysis of Young Ovaries Reveals Candidate Genes Involved in Gamete Formation in *Lantana camara*

**DOI:** 10.3390/plants8080263

**Published:** 2019-08-02

**Authors:** Ze Peng, Krishna Bhattarai, Saroj Parajuli, Zhe Cao, Zhanao Deng

**Affiliations:** Department of Environmental Horticulture, Gulf Coast Research and Education Center, IFAS, University of Florida, 14625 County Road 672, Wimauma, FL 33598, USA

**Keywords:** Lantana, ovary, transcriptome, unreduced gamete

## Abstract

Lantana (*Lantana camara* L., Verbenaceae) is an important ornamental crop, yet can be a highly invasive species. The formation of unreduced female gametes (UFGs) is a major factor contributing to its invasiveness and has severely hindered the development of sterile cultivars. To enrich the genomic resources and gain insight into the genetic mechanisms of UFG formation in lantana, we investigated the transcriptomes of young ovaries of two lantana genotypes, GDGHOP-36 (GGO), producing 100% UFGs, and a cultivar Landmark White Lantana (LWL), not producing UFGs. The *de novo* transcriptome assembly resulted in a total of 90,641 unique transcript sequences with an N50 of 1692 bp, among which, 29,383 sequences contained full-length coding sequences (CDS). There were 214 transcripts associated with the biological processes of gamete production and 10 gene families orthologous to genes known to control unreduced gamete production in *Arabidopsis*. We identified 925 transcription factor (TF)-encoding sequences, 91 nucleotide-binding site (NBS)-containing genes, and gene families related to drought/salt tolerance and allelopathy. These genomic resources and candidate genes involved in gamete formation will be valuable for developing new tools to control the invasiveness in *L*. *camara*, protect native lantana species, and understand the formation of unreduced gametes in plants.

## 1. Introduction

Lantana (*Lantana camara* L., Verbenaceae) is a popular ornamental plant, especially in the subtropical and tropical regions of the world. A survey of the nursery industry in Florida, United States of America (U.S.A.), revealed that hundreds of nurseries and greenhouse growers produced *L. camara*, and the sales of *L. camara* plants contributed more than $40 million a year to the state’s economy [1]. The popularity of *L. camara* is due to its ability to bloom year-round, attract many species of butterflies, tolerate harsh environmental conditions, thrive with low maintenance requirements, and propagate easily [2,3]. The genus *Lantana* is comprised of seven species, with six from South America and one from Ethiopia [4]. Most of the lantana cultivars grown by the ornamental plant industry belong to the species *L. camara. Lantana camara* originated in West Indies and was introduced to the tropical regions of the world by 1900 [5,6]. At present, *L. camara* is found in more than 60 countries under hundreds of cultivar names [7].

*Lantana camara* is also known to be one of the world’s most aggressive invader plant species [2]. As an introduced species in the U.S.A., *L. camara* has escaped from cultivation and established itself in the wild through seed dispersal and hybridization with Florida native lantana species, *Lantana depressa* [8]. Establishment and spread of *L. camara* in Florida has endangered *L. depressa*. The Florida Exotic Pest Plant Council (FLEPPC) has placed *L. camara* as a Category I invasive species in southern, central, and northern Florida (https://www.fleppc.org/). Thus, it is urgent to take actions to control its invasiveness. Considerable efforts have been made to control the spread of invasive *L. camara* using genetic sterilization and the development of sterile cultivars [9,10,11]. A number of sterile cultivars have been released that possess greatly reduced male and female fertility and little potential to cross-pollinate with native lantana. Despite the success, the development of sterile cultivars has been severely complicated by the ability of many *L. camara* cultivars to produce unreduced female gametes (UFGs). The production of UFGs (UFGP) has enabled *L*. *camara* to evolve multiple ploidy levels and many *L*. *camara* triploids and pentaploids to produce viable seeds, as diploids or tetraploids do. Although the formation of unreduced pollen is more frequent in some other plants [12], it is relatively rare in *L. camara* [8]. Therefore, it is important to understand the genetic mechanism(s) underlying the development of UFGs in *L. camara*.

The formation of unreduced gametes (2*n*) is a common mechanism for the generation of polyploids in natural systems [13]. Unreduced gametes have also been employed to engineer sexual polyploidization in several crop species [14,15] and have become a very valuable source of genetic variation for many plant breeding programs. There is on-going research on utilizing unreduced 2*n* gametes to fix heterosis in hybrid seeds. During normal meiosis and gamete formation, the chromosome number in the mother cells is reduced by half. However, 2*n* gametes can be formed due to the first division restitution (FDR) or the second division restitution (SDR) [16]. The molecular mechanisms underlying unreduced gamete formation were studied mainly by analyzing mutants producing unreduced gametes. It was revealed that the formation of unreduced gametes was mainly caused by meiotic defects, including defects in meiotic division, spindle orientation, and cytokinesis [13]. Several genes involved in the formation of unreduced gametes have been identified, mainly in *Arabidopsis*, including *SWITCH1* (*SWI1*)/*DYAD* [17], *CYCA1;2/TAM* [18], *OMISSION OF SECOND DIVISION 1* (*OSD1*) [19], *Arabidopsis Parallel Spindle1* (*AtPS1*) [20], and *Tetraspore/Stud* (*TES/STUD*) [21]. More studies are needed to understand the mechanisms of unreduced gamete formation for utilization in plant breeding and improvement. The *L. camara* plants with the ability to produce UFGs are valuable materials for deepening our understanding of the genetic mechanisms of UFG formation.

*Lantana camara* also possesses a number of other characteristics for plant science research, including tolerance to drought [22,23], salt [24], and allelopathy [25]. These traits imply that *L. camara* may be less prone to diseases or pathogens. The nucleotide-binding site leucine-rich repeat (NBS-LRR) genes play an important role in conferring disease resistance in plants [26]. The identification of genes controlling these characteristics would provide a genomic resource for plant breeding. Nevertheless, only very limited genomic information is available for *L. camara*. Up to date, there is only one transcriptome dataset available at the National Center for Biotechnology Information (https://www.ncbi.nlm.nih.gov/). Very few molecular markers are available for fingerprinting *L. camara* cultivars, differentiating its hybrids with native lantana species, or investigating its population structure and genetic diversity [7]. Therefore, more genomic resources are much needed to expedite the development of sterile *L. camara* cultivars, identify cryptic interspecific hybrids, and understand its invasiveness.

In this study, we applied RNA sequencing to compare the transcriptomes of young ovaries of two *L. camara* genotypes differing in gamete formation. One genotype, Landmark White Lantana (LWL) produces normal reduced (*n*) female gametes (non-UFG-producing or non-UFGP) [27], while the other genotype, GDGHOP-36 (GGO), forms 100% unreduced (*2n*) female gametes (UFG-producing or UFGP) [8]. We revealed candidate genes associated with the formation of UFGs. We further identified gene families related to stress tolerance and disease resistance genes in *L. camara*. Our findings not only significantly enriched the genomic resources of *L. camara*, but also provided insight into the formation of UFGs in *L. camara*. 

## 2. Results

### 2.1. Sample Collection, Illumina Sequencing, and De Novo Assembly

**Figure 1:**
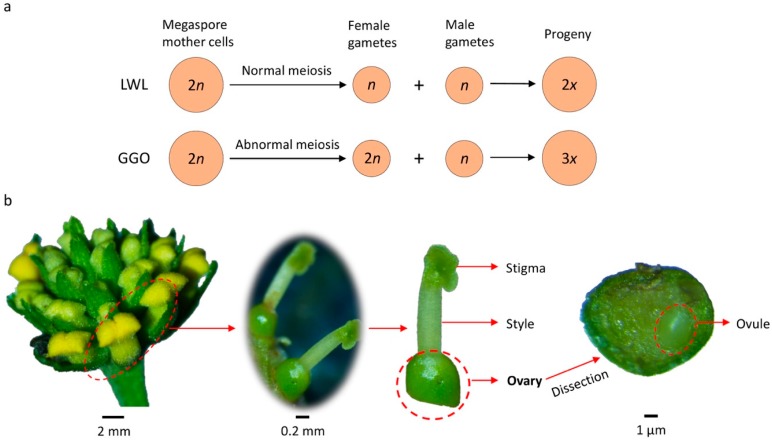
Lantana genotypes and tissues used in this study. (**a**) Schematics showing the differences between Landmark While Lantana (LWL) and GDGHOP-36 (GGO) in the formation of female gametes. (**b**) Demonstration of collecting young ovaries from lantana inflorescences, using a GGO inflorescence as an example.

The commercial cultivar Landmark White lantana (LWL, 2*n* = 2*x* = 22) and a breeding line GDGHOP-36 (GGO, 2*n* = 2*x* = 22) were used for sample collection (Figure 1a). LWL produces normal *n* female gametes. The GGO produces 100% unreduced female gametes (2*n*) [8]. Young ovary tissues of these two genotypes were collected (Figure 1b) for RNA sequencing.

A total of 26,463,996 and 26,540,797 raw read pairs were generated from the transcriptomes of young ovaries of GGO and LWL, respectively. The total sequencing data size was more than 10.6 Gb. After trimming, there were 25,504,854 (96.38%) and 25,554,105 (96.28%) read pairs retained, which were included in downstream analysis. The *de novo* assembly using Trinity generated 112,505 contigs with an N50 of 1787 bp (Table 1). After removing redundancy using CD-HIT-EST program, a unique set of transcript sequences (90,641) were obtained with an N50 of 1692 bp. The length of unique transcripts ranged from 201 bp to 12,218 bp. The length distribution is provided in Figure S1. Among this set of unique transcripts, a total of 48,477 sequences were predicted to contain coding sequences (CDS) and converted to protein sequences using TransDecoder. In total, there were 29,383 transcript sequences containing full-length CDS with the start and stop codon. The size of these full-length transcripts ranged from 327 bp to 12,083 bp, with an N50 of 2206 bp (Table 1).

### 2.2. Functional Annotation of the Unique Transcripts

**Figure 2:**
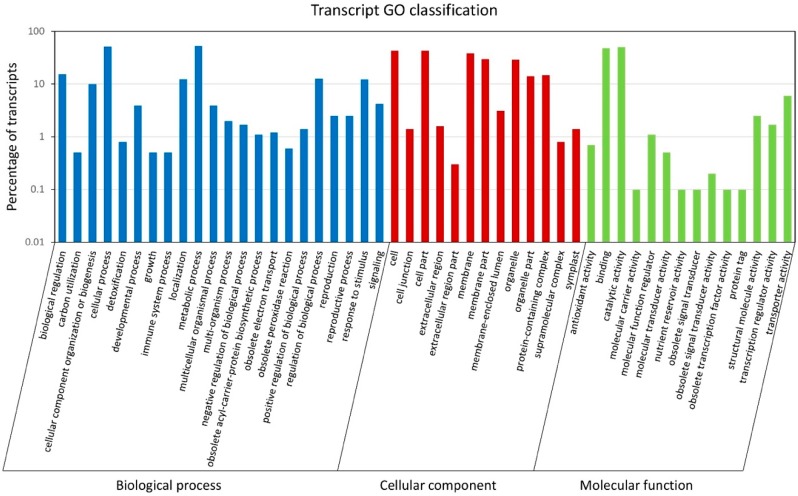
Gene ontology classification of *Lantana camara* unique transcripts. The histogram shows the classification of unique transcripts under three categories, including biological process (blue), cellular component (red), and molecular function (green). The y-axis indicates the number of transcripts (log_10_ scale).

**Figure 3:**
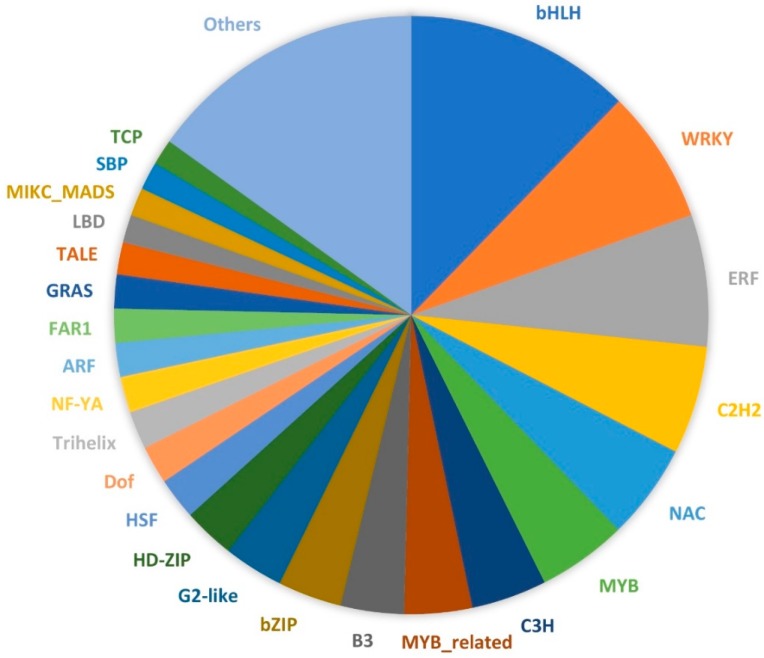
Transcription factors identified from the young ovary transcriptomes of *Lantana camara*. Different colors represent different transcription factor gene families.

The 90,641 unique transcript sequences were annotated by comparing them to several major databases (Tables S1 and S2). A large portion of the sequences (57,191; 63.09%) had hits in the non-redundant protein (NR) database, followed by Swiss-Prot (43,895; 48.43%) and the nucleotide (NT) databases (39,776; 43.88%). A total of 5650 Gene Ontology (GO) terms and 3836 Kyoto Encyclopedia of Genes and Genomes Ontology (KO) terms were assigned to 35,765 and 15,950 transcripts, respectively. The GO terms were classified into three categories, including biological process, cellular component, and molecular function (Figure 2). Within the biological process category, most transcripts were assigned to metabolic process (53%), cellular process (50.9%), and biological regulation (15.4%). For the cellular component class, more transcripts were associated with the cell (42.9%), cell part (42.5%), and membrane (38.3%). In terms of molecular function, most of the transcripts were involved in catalytic activity (50.4%) and binding (48.3%). The GO terms associated with gamete formation and their child terms were further investigated in these annotated transcript sequences. A total of 214 sequences were associated with gamete formation-related biological processes, including cell cycle-related processes, cytokinesis, chromosome organization and segregation, and gamete generation (Table 2 and Table S3). By comparing the transcript sequences to the PlantTFDB v4.0, a total of 925 transcription factor (TF)-encoding sequences were identified and assigned to 49 TF families (Table S4). Among these TF families, the bHLH family of TFs (114) were the most predominant, followed by WRKY (67), ERF (66), C2H2 (54), NAC (48), and MYB (45) (Figure 3).

### 2.3. Gene Expression in the Ovaries of GGO and LWL Lantana

**Figure 4:**
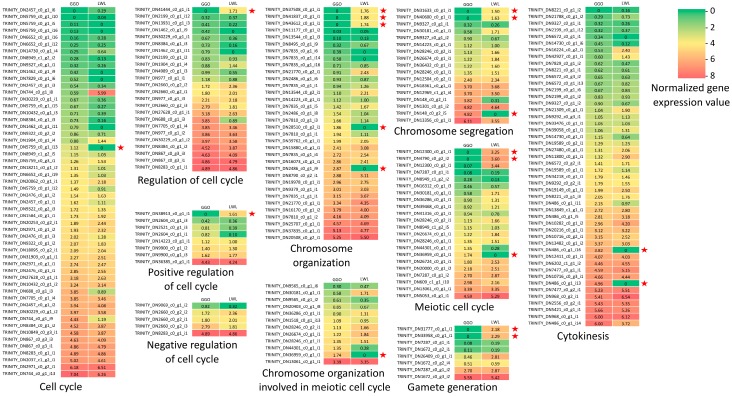
Heat maps of gene expressions for genes potentially affecting gamete formation in LWL and GGO. The expression values are log2(x + 1) transformed. Left column indicates expression values in GGO. Right column indicates expression values in LWL. The normalized expression values vary from 0 (green) to 8 (red). The 18 transcripts that were expressed (expression value ≥ 1) in one genotype but not expressed in the other are labelled with a star sign.

Since GGO lantana produced unreduced gametes and LWL lantana produced normal reduced gametes, their gene expression profiles in ovaries were further investigated. Overall, there were 64,397 transcripts actively expressed (fragments per kilobase of transcript per million mapped reads or FPKM ≥ 1) in the ovaries of at least one genotype, including 52,265 transcripts from GGO and 49,169 transcripts from LWL (Table S2). Genes that were actively expressed (FPKM ≥ 1) in one genotype and not expressed (FPKM = 0) in the other were specifically examined. In total, 5224 transcripts were actively expressed in the ovaries of GGO, but not expressed in the ovaries of LWL; 4314 transcripts were actively expressed in the ovaries of LWL, but not expressed in the ovaries of GGO. GO enrichment analysis revealed no significantly enriched GO terms for the transcripts expressed in GGO but not in LWL. However, there were 43 GO terms significantly enriched for the transcripts expressed in LWL but not in GGO (Table S5). This GO enrichment analysis showed that most or all the transcripts associated with those enriched GO terms were not expressed in GGO (unreduced gamete production), but actively expressed in LWL (normal reduced gamete production) (Table S5). Specifically, several GO terms related with telomeres were among those GO terms, including the positive regulation of establishment of protein localization to the telomere (GO:1904851), positive regulation of telomere maintenance via telomerase (GO:0032212), and positive regulation of telomerase RNA localization to the Cajal body (GO:1904874). Out of the five total transcripts associated with these telomere-related activities in the whole transcriptome, four of them were not expressed in GGO, whereas all five transcripts were actively expressed in LWL. This result showed the telomere-related activities were undermined in GGO compared with LWL. Further, the expressions of the transcripts associated with gamete formation from GGO and LWL were retrieved to construct heat maps (Figure 4). In general, GGO and LWL had similar expressions for most of the transcripts. However, there were 18 transcripts only expressed in one genotype but not in the other genotype. These genes were potentially associated with the differences in gamete formation in the two genotypes.

### 2.4. Lantana Gene Families

To identify lantana gene families associated with unreduced gamete formation, drought tolerance, salt tolerance, and allelopathy, publicly available genes were searched in the literatures and UniProtKB database. In total, we collected 13 genes that were known to be involved in unreduced gamete production in *Arabidopsis*. In addition, 260 genes related with drought tolerance, 494 genes related with salt tolerance, and four genes related with allelopathy were collected from the UniProtKB database (Table S6). Their orthologs in lantana were identified through gene family analysis. Finally, there were 10 lantana gene families related with unreduced gamete production that were identified, including orthologs of *MPK4*, *PS1*, *JASON*, *TES*, *INCENP*, *RBR*, *OSD1*, *RMS/ESP1*, *SPO11-1*, and *MKK6/ANQ1*. Further investigation of gene expressions revealed that these orthologs in lantana had similar expression levels between GGO and LWL, with no obvious difference. We found 291 gene families related with drought tolerance and/or salt tolerance, in which 95 gene families were involved in both drought tolerance and salt tolerance (Table S7). The lantana orthologs of the four allelopathy genes were also identified (Table S7). Among the identified gene families, one gene family (OG1.5_1078) orthologous to *MPK4* was related with both drought/salt tolerance and unreduced gamete production.

### 2.5. Identification and Classification of NBS Genes

**Figure 5:**
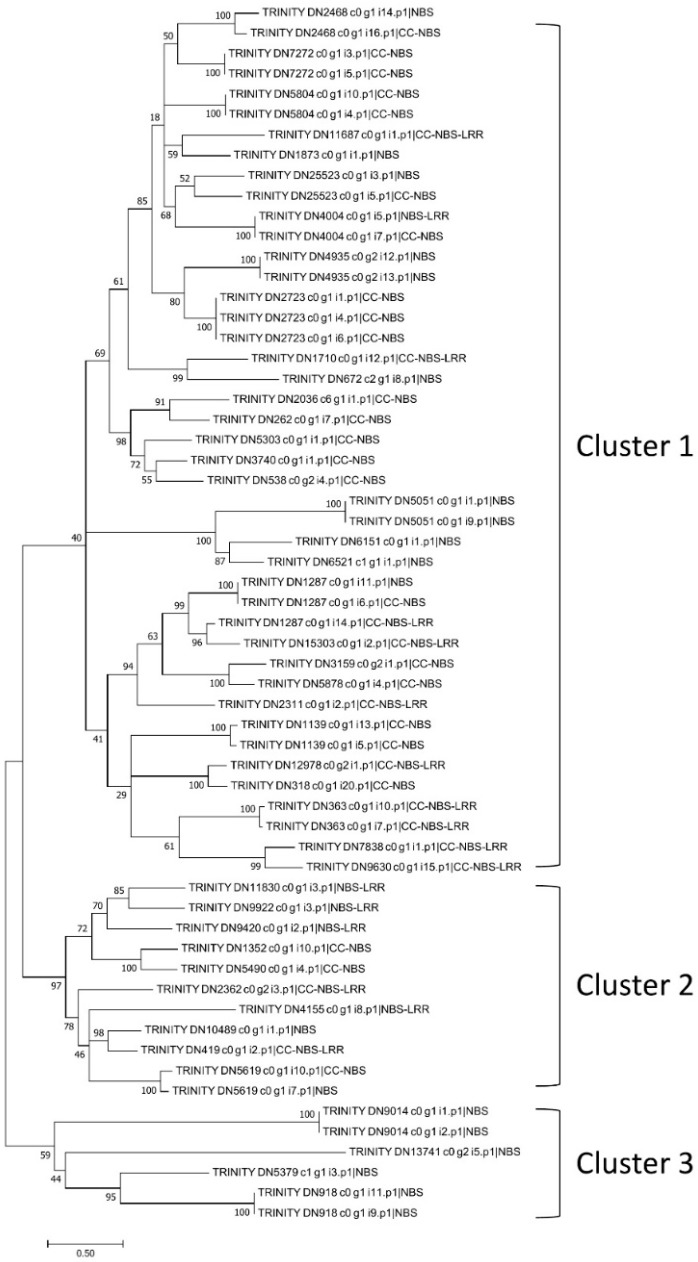
Phylogenetic tree of *Lantana camara* NBS genes. Three clusters can be recognized in the phylogenetic tree: Cluster 1 and Cluster 2 both containing CC-type NBS genes, and Cluster 3 not containing CC-type NBS genes.

As lantana has high levels of resistance to common bacterial and fungal diseases, NBS genes were analyzed using the protein sequences (with complete CDS) predicted by TransDecoder. After removing redundancy using CD-HIT, a total of 19,686 lantana proteins were analyzed. First, 89 NBS proteins were identified using the hidden Markov model (HMM) profile of the nucleotide-binding, apoptotic protease-activating factor-1, R protein, and Caenorhabditis elegans death-4 protein (NB-ARC) domain. After applying a Lantana-specific HMM profile, two more NBS proteins were identified. Overall, 91 NBS genes were identified (Table S8). The NBS genes were further classified based on the presence of the nucleotide-binding site (NBS), Toll/Interleukin-1 receptor (TIR), coiled coil (CC), and leucine-rich repeat (LRR) domains (Table 3). Most of the identified NBS proteins were NBS type (43), followed by CC-NBS type (29), CC-NBS-LRR type (12), and NBS-LRR type (7). The TIR domain was not detected within these NBS proteins. To investigate the genetic relationship of the NBS genes, 60 of them with a complete NBS domain were used for constructing a phylogenetic tree. Three distinct clusters were generated (Figure 5). Cluster 1 contained more NBS proteins (43) than Cluster 2 (11) and Cluster 3 (6). Both Cluster 1 and Cluster 2 contained CC- type NBS proteins, while Cluster 3 NBS proteins were non-CC type.

### 2.6. Discovery of Simple Sequence Repeats, Single Nucleotide Polymorphisms, and Insertion or Deletions

Using the unique transcript sequences, a total of 10,190 simple sequence repeats (SSRs) were discovered, including 7886 di-nucleotide SSRs, 2118 tri-nucleotide SSRs, 101 tetra-nucleotide SSRs, 30 penta-nucleotide SSRs, and 55 hexa-nucleotide SSRs (Table 4). In general, the number of SSRs decreased with the increase of the nucleotide number of the SSR repeat unit. Among the 97 repeat motifs identified, the AG/CT motif (43.07%) was the most abundant, followed by AT/AT motif (18.95), and AC/GT motif (15.19%) (Table S9). Primers were successfully designed for 9513 SSRs (93.36%) (Table S10).

After variant calling using Samtools and filtering based on read depth, a total of 165,229 single nucleotide polymorphisms (SNPs) and 9984 insertion or deletions (indels) between GGO and LWL were identified (Table 5 and Table S11). Most of these SNPs (84.88%) and indels (79.13%) were heterozygous variants, in which one genotype was heterozygous (0/1) and the other genotype was homozygous (0/0 or 1/1). A relatively small proportion of them were homozygous variants, in which both genotypes were homozygous (Table 5). The effects of these variants were annotated using SNPeff. It revealed that a large proportion of the SNPs were predicted to have a low impact on gene functions, including synonymous variants (51,878; 33.67%) and those located within untranslated (UTR) regions (56,103; 36.42%), providing no change to coding regions (Table 6). Still, there were 45,326 (29.42%) missense variants, leading to codon change, and a small proportion of SNPs (752; 0.49%) that changed start or stop codons, which could potentially influence gene functions. Similarly, for indels, most of them were located within either 3′ UTR (3497; 39.34%) or 5′ UTR (2720; 30.60%) with a low impact on gene functions. A smaller proportion of them were predicted to have significant impacts on gene functions. The identified SSRs, SNPs, and indels provided a good resource of functional markers in lantana.

Further screening of the identified SNPs and indels revealed 83 SNPs and 7 indels located within transcripts that were associated with gamete production or orthologous to genes known to control unreduced gamete production (Table S12). While most of these variants were predicted to have a low impact on gene functions, there were two indels leading to an in-frame change of CDS length. One was in a gene (TRINITY_DN19149_c0_g1_i1) encoding a guanosine triphosphate-binding (GTP-binding) protein, while the other was in a gene (TRINITY_DN14223_c0_g1_i1) orthologous to *RMS/ESP1*. Additionally, 25 SNPs were missense variants changing amino acid sequences. These variants that could potentially influence gene functions are good candidates for future identification of mutations leading to unreduced gamete formation in GGO.

## 3. Discussion

As an important ornamental plant and an invasive species, *L. camara* is understudied and has very limited genomic resources. The development of sterile triploid lantana cultivars is an effective approach to controlling their invasiveness [9]. However, this is hindered by the formation of UFGs frequently observed in *L. camara* [8]. Currently, little is known about the underlying genetic mechanisms of UFG formation in lantana. Our study investigated the transcriptome profiles of young ovaries of a lantana line producing UFGs and another cultivar producing normal gametes. The transcriptome analysis significantly enriched the genomic resources of lantana by contributing transcript sequences and molecular markers in a transcriptome-wide manner. The comparative analysis between the two genotypes revealed genes associated with female gamete production, as well as those orthologous to genes known to control unreduced gamete production. The development of molecular markers located within those genes and the markers that potentially change gene functions provided good candidates for discovering the mutations controlling the formation of UFGs. In addition, the identification of disease resistance genes and orthologous gene families associated with stress (drought/salt) tolerance and allelopathy would facilitate understanding the special characteristics of lantana and provide a genomic resource for plant breeding. Results from this study help us gain very valuable insight into the genetic basis of UFG formation in *L. camara* and may enable the development of novel genetic tools for manipulating gamete formation in plant breeding, genetic improvement, and invasive species management.

The ovary is an organ where female gametes are produced. There have been a few transcriptome studies performed on ovary tissues to investigate gene regulations of fruit development in tomato (*Solanum lycopersicum*) [28,29], wild tomato (*Solanum pimpinellifolium*) [30], and rice (*Oryza sativa*) [31], to elucidate carpel fusion mechanisms in maize (*Zea mays*), to find genes regulating embryo and pod development in peanut (*Arachis hypogaea*) [32], to find genes responsive to freezing stress in almond (*Prunus dulcis* Mill.) [33], and to identify genes controlling ovary coloration in Asiatic hybrid lilies (*Lilium* spp.) [34]. By focusing on the 29,383 transcripts containing full-length CDS, the N50 increased to 2206 bp. The unique transcript sequences and those containing full-length CDS provide a valuable resource for further studies of genes expressed in ovaries of lantana. Functional annotation revealed that most of those sequences had significant hits in the five major databases (NR, NT, Kyoto Encyclopedia of Genes and Genomes or KEGG, GO, and Swiss-Prot) (Table S1), which facilitated the identification of transcripts associated with gamete formation.

Gamete formation involves a series of cell divisions, and meiosis is particularly critical for the production of gametes with reduced chromosome numbers. Defects in these cell cycles, early meiotic events, spindle orientation, or cytokinesis can all lead to the formation of unreduced gametes [13]. Through gene family analysis, there were 10 lantana gene families that were orthologous to genes known to control unreduced gamete production in *Arabidopsis*. Furthermore, a total of 214 lantana transcript sequences were associated with gamete production based on annotated functions. These genes can be further studied in the future to understand the UFG formation in lantana. They can also potentially serve as candidates for gene editing. Among the 925 identified TF-encoding sequences, the most abundant TF family bHLH may play an important role during the development of lantana ovaries. As supported by studies in *Arabidopsis*, bHLH TFs, such as *CRABS CLAW* (*CRC*), *SPATULA* (*SPT*), and *HECATE* (*HEC*), were reported to regulate the development of gynoecium, the female reproductive organ [35,36].

Direct comparison of the transcriptome profiles of young ovaries between two lantana genotypes differing in gamete production enabled a glance at the expressions of genes related with gamete production. To identify the genes that are actively expressed (likely to be functional in ovaries) in one genotype and not expressed (unlikely to be functional in ovaries) in the other, we specifically focused on those with an FPKM value ≥1 in one genotype and FPKM = 0 in the other genotype. Overall, there were 5224 transcripts actively expressed in GGO and not expressed in LWL. As LWL forms reduced gametes, the absence of these gene expressions in LWL may indicate that they are not required for normal gamete production. However, we cannot exclude the possibility that some genes related with UFG formation are among these 5224 genes. GO enrichment analysis showed that no GO terms were enriched for these genes, whereas there were 43 GO terms enriched in the 4314 transcripts actively expressed in LWL and not expressed in GGO. These genes were likely playing normal functions in the ovaries of LWL, but not in the ovaries of GGO due to the absence of expressions. They potentially included the genes whose absence of expressions were caused by the formation of UFGs or led to the formation of UFGs in GGO. Among these 4314 transcripts, there were 11 transcripts associated with gamete formation-related biological processes. Moreover, we found three enriched GO terms related with the telomere. During meiosis, chromosome pairing at prophase is required for subsequent chromosome segregation that reduces the chromosome number before gamete formation [37]. The rapid prophase chromosome movement is led by telomeres, which is important to chromosome pairing and synapsis. The telomeres cluster prior to the initiation of synapsis [38]. Therefore, the disturbed telomere migration could lead to failures of synapsis and chiasma formation [39]. Out of the total five transcripts associated with these three telomere-related GO terms, four were not expressed in GGO, but all five were actively expressed in LWL, indicating likely that telomere activities during meiosis is disrupted in GGO. This may imply a very important role of telomeres in unreduced gamete formation. These 15 transcripts seem to be good candidates for the future study of gamete production in lantana (and other plants).

Since the lantana genome reference is not available, we annotated the effects of sequence variants using the transcriptome as a reference. Particularly, we focused on the polymorphisms in genes associated with gamete production and the genes that were orthologs to those known to control unreduced gamete production in other plants. Toward this end, we identified 83 SNPs and seven indels in 19 genes associated with gamete formation and nine orthologs likely controlling unreduced gamete formation. Variant annotations indicated that 25 SNPs and two indels out of these variants likely have an impact on gene functions. More studies are needed to identify the causal mutations leading to UFG formation in lantana.

Toward further understanding and better utilization of the special characteristics of lantana, including its disease resistance, drought/salt tolerance, and allelopathy, we discovered the putative disease resistance (NBS) genes, and gene families related with drought/salt tolerance and allelopathy for the first time in lantana. In total, we identified 91 NBS genes and three gene clusters based on phylogenetic analysis. Similarly, based on a previous study that clustered NBS genes from multiple species, three major groups were obtained, including two groups of the non-TIR NBS genes and one TIR group [40]. However, no TIR domain was identified within the NBS genes in this study, which was likely due to the incomplete coverage of NBS genes or a specific feature of lantana NBS genes. In addition, we identified 291 gene families related to drought/salt tolerance and four gene families related to allelopathy, which enriched the gene pool of stress tolerance in plants and can be further explored in the future.

Molecular markers are an important genomic resource that have many applications, such as cultivar fingerprinting, identification of cryptic interspecific hybrids, genetic mapping, genetic diversity analysis, and phylogenetic analysis [41]. A major issue caused by the invasiveness of *L. camara* is its hybridization with *L. depressa*, making *L. depressa* an endangered species [42]. Molecular markers based on SSRs and indels can be easily developed into PCR-based genotyping tools, which will be invaluable to many applications, including identifying cryptic hybrids resulting from unintended natural pollination or crossing between *L*. *camara* and *L*. *depressa* and protecting the native lantana species in ecological conservation and restoration. In this study, we catalogued a total of 9513 SSRs in lantana, with primers designed for each. Moreover, we identified 165,229 SNPs and 9984 indels that were polymorphic between LWL and GGO, and further annotated their effects. Some of the variations could lead to codon changes, including start or stop codons, and they may significantly impact gene functions and lead to changes in lantana phenotype.

## 4. Materials and Methods

### 4.1. Plant Materials, RNA Extraction, and Sequencing

The commercial cultivar Landmark White lantana (LWL, 2*n* = 2*x* = 22) and a breeding line GDGHOP-36 (GGO, 2*n* = 2*x* = 22) were used in this study. Plants were grown in plastic containers and maintained in the greenhouse at the University of Florida’s Gulf Coast Research and Education Center (Wimauma, FL, USA). We used the ovaries from those flowers that were not open. Young ovary tissues (small, green, and hard) of these two genotypes were collected. First, the petals and upper part of the pistils (stigma and style) were removed. Then forceps were used to excise the ovary out of the pistil. The ovary tissues were frozen in liquid nitrogen immediately. Approximately 40 young ovaries from each genotype were collected and pooled. Ovary samples were shipped to Beijing Genomics Institute (BGI, Shenzhen, China). RNA was extracted using the RNeasy Plant Mini Kit (Qiagen, Hilden, Germany). The quality and quantity of RNA were evaluated spectrophotometrically using the NanoDrop (Thermo Scientific, Wilmington, USA) and Qubit 2.0 (Invitrogen, Waltham, USA). The RNA integrity value was measured using a Bioanalyzer 2100 (Agilent Technologies, Santa Clara, USA). The cDNA libraries were constructed following the same method as previously described [43]. Sequencing was performed on an Illumina HiSeq 2000 platform (100 bp paired-end reads).

### 4.2. Sequence Trimming, De Novo Assembly, and Clustering

The raw sequencing reads were trimmed using Trimmomatic (ILLUMINACLIP:adapters.fasta:2:30:20 LEADING:3 TRAILING:3 SLIDINGWINDOW:4:15 MINLEN:50) [44]. Read quality was assessed using FastQC [45]. The trimmed reads were *de novo* assembled using Trinity (--CPU 25 --min_kmer_cov 2) [46]. The redundancy of assembled sequences was removed using CD-HIT-EST (-c 0.95 -n 9 -T 0 -M 0 -r 1) [47]. Only sequences more than 200 bp in length were subjected to downstream analysis.

### 4.3. Function Annotation

The unique transcript sequences obtained from CD-HIT-EST were compared to several major databases, including the non-redundant protein (nr), non-redundant nucleotide (nt) databases from NCBI (https://www.ncbi.nlm.nih.gov/), SWISS-PROT database (https://www.uniprot.org/), and Kyoto Encyclopedia of Genes and Genomes (KEGG) database (http://www.genome.jp/kaas-bin/kaas_main), using BLAST under E-value cutoff 1e-05. Gene ontology (GO) terms were assigned to sequences using Blast2Go (-v -annot -dat -img -ips ipsr -annex -goslim) [48]. The GO annotation was further classified using WEGO software [49]. Transcription factors (TFs) were predicted using the Plant Transcription Factor Database (PlantTFDB) v4.0 (http://planttfdb.cbi.pku.edu.cn/prediction.php) with default settings. TransDecoder was used to predict coding sequences (CDS) within transcript sequences and convert them into protein sequences (https://github.com/TransDecoder/TransDecoder). The homology searches using SWISS-PROT and Pfam databases were integrated into CDS selection following the suggestion of TransDecoder. Redundancy of converted proteins was further removed using CD-HIT (-c 0.95 -n 5 -T 0 -M 0 -d 0) for gene family analysis.

### 4.4. Mapping and Gene Expression

The trimmed reads of LWL and GGO were aligned to the unique transcript sequences obtained from CD-HIT-EST using BWA v0.7.17 (bwa mem) [50]. Uniquely mapped reads were retrieved by filtering off reads with a mapping quality of zero and the “XA:Z” tag. Only uniquely mapped reads were considered for calculating gene expressions using GFOLD v1.1.4 [51]. To obtain a heat map of gene expressions potentially related with gamete formation, the gene expression values obtained from GFOLD were transformed using log2(x + 1) method. The heat maps were constructed using Microsoft Excel^TM^ Conditional Formatting function.

### 4.5. Lantana Gene Families Involved in Unreduced Gamete Production, Drought Tolerance, Salt Tolerance, and Allelopathy

Genes known to control the unreduced gamete production in *Arabidopsis* were searched for in the literatures [13,52,53]. Publicly available genes related to drought tolerance, salt tolerance, and allelopathy were searched using these key words at UniProtKB database (https://www.uniprot.org/). Only manually annotated and reviewed proteins were kept for analysis. The lantana protein sequences and collected protein sequences were compared with each other using the ALL-AGAINT-ALL Blastp (E-value 1 × 10^−5^). The orthologous gene families were assigned using OrthoMCL [54] (inflation value 1.5).

### 4.6. Identification of NBS Genes and Phylogenetic Analysis

The protein sequences predicted using TransDecoder, after removing redundancy using CD-HIT, were used for the identification of nucleotide-binding site (NBS)-containing genes. The protein sequences were first screened using “hmmsearch” and the hidden Markov model (HMM) profile of the NBS (PF00931) [55] under E-value 1 × 10^−4^. PfamScan was used to further confirm the presence of the NBS domain (E-value 1 × 10^−4^). Further, a Lantana-specific NBS domain HMM profile was constructed using high quality hits from “hmmsearch” (E-value 1 × 10^−60^). These protein sequences were aligned using ClustalW2 [56], which was further used for constructing the Lantana-specific HMM profile with “hmmbuild.” The original protein sequences were re-searched using this HMM profile (hmmsearch) and confirmed with PfamScan. The NCBI Conserved Domains tool was used to search for the presence of the Toll/Interleukin-1 receptor (TIR), coiled coil (CC), and leucine-rich repeat (LRR) domains with default settings. For the genes with a complete NBS domain, the NBS domain sequences were extracted and used to construct a phylogenetic tree with MEGA7 [57].

### 4.7. Identification and Annotation of SSR, SNP, and Indel Variants

Simple sequence repeats (SSRs) identification and primer design were performed using MISA and Primer3 [58]. For single nucleotide polymorphism (SNP) and insertion or deletion (indel) identification, the mapping file (SAM) containing only uniquely mapped reads were used. Variants were called using Samtools v1.9 [59] (-q 30 -Q 20). Both SNPs and indels were filtered based on the following criteria: at least four reads covering the position for a homozygous genotype; at least two reads covering both the reference and alternate allele for a heterozygous genotype. SNPeff was used for annotating the effects of SNPs and indels [60]. A new database was built using the lantana transcript file and the “General Feature Format (GFF)” file obtained from TransDecoder.

## 5. Conclusions

This study investigated the transcriptomes of young ovaries of two lantana genotypes, one forming normal reduced gametes and the other forming UFGs. The ovary transcriptome sequences provided an excellent resource for studying the reproductive development and gamete production in lantana. The transcript sequences, TFs, molecular markers, NBS genes, and gene families related with stress tolerance can become an invaluable resource for genetic research in lantana. These genomic resources will expedite the development of sterile lantana cultivars and further our understanding of the genetic mechanisms controlling gamete formation in lantana and other plants.

## Figures and Tables

**Table 1:** Summary statistics of *Lantana camara* ovary transcriptome assembly using Trinity and clustering using CD-HIT-EST.

	Trinity	CD-HIT-EST	TransDecoder (Full-Length)
No. of sequences	112,505	90,641	29,383
Total length (bp)	126,460,570	94,042,530	57,219,462
Average length (bp)	1124	1037.53	1947.40
N50 (bp)	1787	1692	2206
Minimum length (bp)	201	201	327
Maximum length (bp)	12,218	12,218	12,083

**Table 2:** Summary of *Lantana camara* transcripts associated with gamete formation-related biological processes.

Biological Process	Gene Ontology (Including Child Terms)	No. of Transcripts
Cell cycle	GO:0007049	57
Regulation of cell cycle	GO:0051726	25
Positive regulation of cell cycle	GO:0045787	16
Negative regulation of cell cycle	GO:0045786	5
Meiotic cell cycle	GO:0051321	22
Cytokinesis	GO:0000910	53
Chromosome organization	GO:0051276	34
Chromosome segregation	GO:0007059	18
Chromosome organization involved in meiotic cell cycle	GO:0070192	16
Gamete generation	GO:0007276	8
Total (unique)		214

**Table 3:** Classification of NBS genes in *Lantana camara* ovary transcriptomes.

NBS Gene Class	Number of Genes
CC-NBS-LRR	12
CC-NBS	29
NBS-LRR	7
NBS	43
Total	91

Note: “CC” indicates the coiled coil domain, “NBS” indicates the nucleotide-binding domain, and “LRR” indicates the leucine-rich repeat domain.

**Table 4:** Summary statistics of *Lantana camara* simple sequence repeats (SSRs) identified and primers designed based on the SSRs.

SSRs	Repeat Motif Length					Total
	Di-	Tri-	Tetra-	Penta-	Hexa-	
SSRs identified in transcript assembly	7886	2118	101	30	55	10,190
SSRs designed with primers	7325	2007	97	30	54	9513

**Table 5:** Summary statistics of identified SNPs and indels.

SNP Type	SNP Number	INDEL Number	GGO Genotype	LWL Genotype
Homozygous	11,865	1014	0/0	1/1
	13,115	1070	1/1	0/0
Heterozygous	54,628	3052	0/0	0/1
	74,210	4194	0/1	0/0
	6494	377	0/1	1/1
	4917	277	1/1	0/1
Total	165,229	9984		

Note: “0/0” indicates a homozygous genotype with the reference allele, “1/1” indicates a homozygous genotype with the alternate allele, and “0/1” indicates a heterozygous genotype.

**Table 6:** Summary of the predicted effects of SNP and indel variants identified in two *Lantana camara* differing in female gamete formation.

Variant Effects	Count	Percentage (%)	Variant Type
Synonymous variant	51,878	33.67	SNP
Missense variant	45,326	29.42	SNP
3′ UTR variant	31,145	20.22	SNP
5′ UTR variant	21,390	13.88	SNP
5′ UTR premature start codon gain variant	3568	2.32	SNP
Stop gained	529	0.34	SNP
Stop lost	119	0.08	SNP
Stop retained variant	62	0.04	SNP
Start lost	42	0.03	SNP
Initiator codon variant	9	0.01	SNP
3′ UTR variant	3497	39.34	Indel
5′ UTR variant	2720	30.60	Indel
Frameshift variant	1029	11.58	Indel
Conservative inframe deletion	516	5.80	Indel
Conservative inframe insertion	471	5.30	Indel
Disruptive inframe deletion	309	3.48	Indel
Disruptive inframe insertion	245	2.76	Indel
Stop gained	49	0.55	Indel
Stop lost	30	0.34	Indel
Start lost	23	0.26	Indel

## Supplementary Materials

Supplementary materials can be found at https://www.mdpi.com/2223-7747/8/8/263/s1. Figure S1: The length distribution of *Lantana camara* unique transcripts. Table S1: Transcript homology searches against major databases. Table S2: Annotation and expression values of transcripts. Table S3: *Lantana camara* ovary transcripts associated with gamete formation-related biological processes and their expression values. Table S4: Transcription factors predicted from the unique transcript sequences of *Lantana camara*. Table S5: Gene ontology terms enriched in transcripts only expressed in *Lantana camara* cultivar Landmark White lantana (LWL). Table S6: Published genes used for gene family analysis in this study. Table S7: *Lantana camara* gene families related with unreduced gamete formation, drought tolerance, salt tolerance, and allelopathy. Table S8: Identified NBS genes from *Lantana* ovary transcriptome data. Table S9: SSR motif types and numbers identified in *Lantana camara* ovary transcript assembly. Table S10: Primers designed for SSRs identified in *Lantana camara* ovary transcript assembly. Table S11: Identified SNPs, Indels and their annotated effects. Table S12: SNPs and Indels located within *Lantana* transcripts potentially involved in gamete production. Raw sequencing reads have been deposited in the Sequence Read Archive (SRR9734879, SRR9734880). The assembled transcripts were deposited in GenBank (TSA accession number GHRO00000000).
