# Supplementary material for: Transcriptome Analysis of Young Ovaries Reveals Candidate Genes Involved in Gamete Formation in Lantana camara
Source: Plants (Basel). 2019 Aug 2;8(8):263. doi: 10.3390/plants8080263 (PMC6724078; doi:10.3390/plants8080263)
Supplement: Supplementary file 1 [file plants-08-00263-s001.zip › Supplementary files.docx]

**Supplementary Tables**

**Supplementary Table 1.** Transcript homology searches against major databases.

**Supplementary Table 2.** Annotation and expression values of transcripts.

**Supplementary Table 3.** *Lantana* *camara* ovary transcripts associated with gamete formation-related biological processes and their expression values.

**Supplementary Table 4.** Transcription factors predicted from the unique transcript sequences of *Lantana camara*.

**Supplementary Table 5.** Gene ontology terms enriched in transcripts only expressed in *Lantana* *camara* cultivar Landmark White lantana (LWL).

**Supplementary Table 6.** Published genes used for gene family analysis in this study.

**Supplementary Table 7.** *Lantana camara* gene families related with unreduced gamete formation, drought tolerance, salt tolerance, and allelopathy.

**Supplementary Table 8.** Identified NBS genes from *Lantana* ovary transcriptome data.

**Supplementary Table 9.** SSR motif types and numbers identified in *Lantana* *camara* ovary transcript assembly.

**Supplementary Table 10.** Primers designed for SSRs identified in *Lantana* *camara* ovary transcript assembly.

**Supplementary Table 11.** Identified SNPs, Indels and their annotated effects.

**Supplementary Table 12.** SNPs and Indels located within *Lantana* transcripts potentially involved in gamete production.

**Supplementary Figures**

**Supplementary Figure 1.** The length distribution of *Lantana* *camara* unique transcripts.
