# Supplementary figures and images for: Transcriptome Analysis of Young Ovaries Reveals Candidate Genes Involved in Gamete Formation in Lantana camara
Source: Plants (Basel). 2019 Aug 2;8(8):263. doi: 10.3390/plants8080263 (PMC6724078; doi:10.3390/plants8080263)

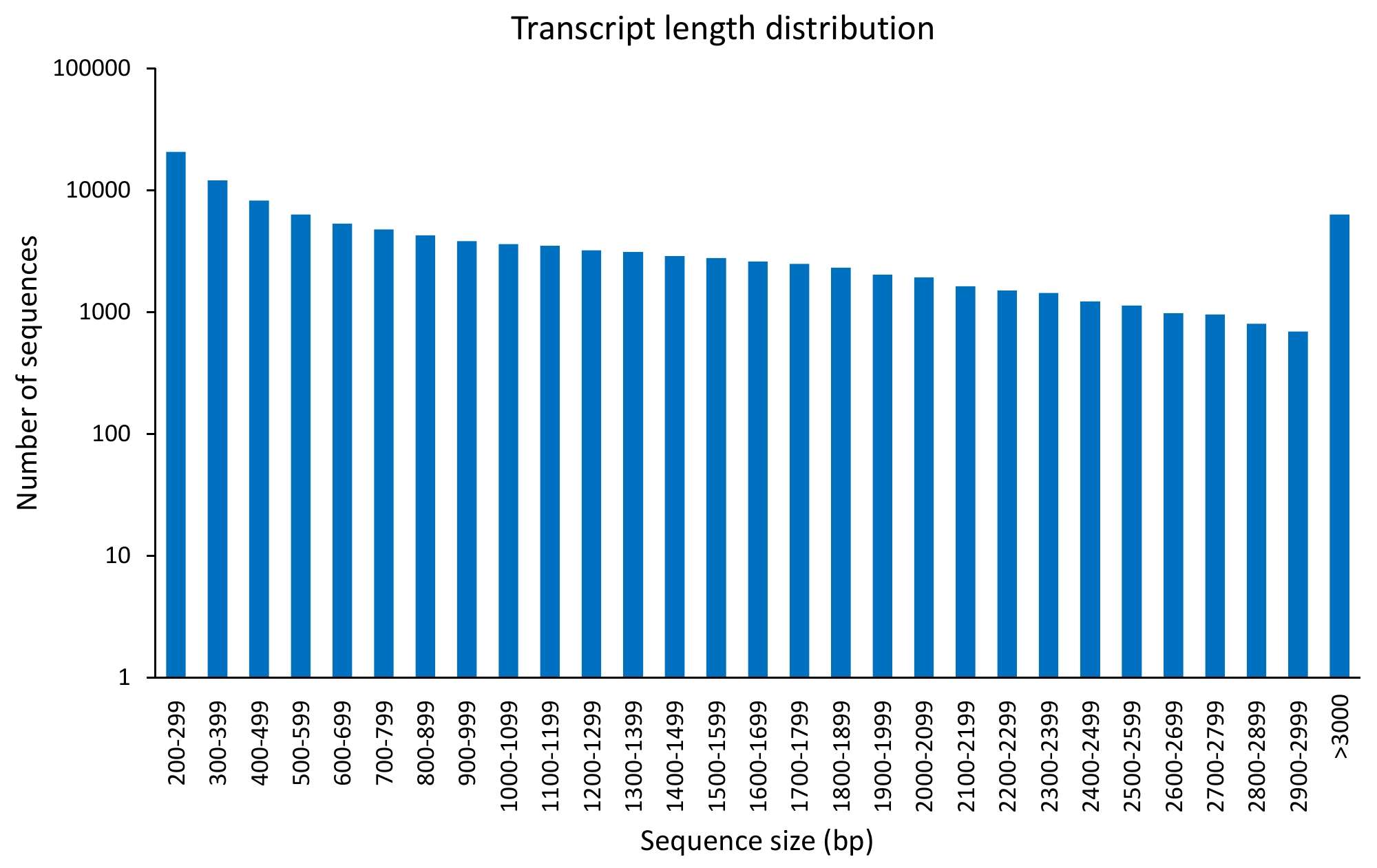

Supplement: Supplementary file 1 [file plants-08-00263-s001.zip › Supplementary figure 1.jpg]
